# A Novel Transcatheter Device to Treat Calcific Aortic Valve Stenosis: An Ex Vivo Study

**DOI:** 10.1007/s13239-025-00774-1

**Published:** 2025-02-05

**Authors:** Francesca Perico, Eleonora Salurso, Fabio Pappalardo, Michal Jaworek, Enrico Fermi, Maria Chiara Palmieri, Flavius Constantin Apostu, Riccardo Vismara, Marco Vola

**Affiliations:** 1 Department of Electronics, Information and Bioengineering, Politecnico di Milano, Via Golgi 39, 20133 Milan, Italy; 2 AorticLab S.r.l., Bioindustry Park, Colleretto Giacosa, Turin, Italy; 3 Hôpital Cardiologique et Pneumologique Louis-Pradel, Hospices Civils de Lyon, Bron, France

**Keywords:** Aortic valve stenosis, Transcatheter debridement device, Ultrasound shockwave, Ex-vivo set up, Human heart

## Abstract

**Purpose:**

Aortic valve stenosis (AVS) is the most common valvular disease in developed countries. Surgical or transcatheter bioprosthetic aortic valve (AV) replacement is the standard treatment for severe AVS. However, bioprostheses are prone to structural degeneration. Hence, in terms of lifetime management, there is a need for therapies that can postpone AV replacement. With the aim of fragmenting calcifications and restoring AV leaflets flexibility, a new transcatheter debridement device (TDD) exploiting ultrasound is under development. We performed an ex-vivo study on human hearts to quantify how TDD treatment affects stenotic AVs hemodynamic. Additionally, a qualitative histological analysis was performed to assess TDD’s impact on AV leaflets.

**Methods:**

Three human hearts affected by AVS were characterized pre- and post-treatment in an ex-vivo beating heart simulator. To replicate physiological flowrates, a pulsatile pump was connected to the left ventricle, while a systemic impedance simulator connected to the aortic root and a reservoir connected to the left atrium closed the hydraulic circuit. Transvalvular pressure drop (ΔPsys), backflow volume, and effective orifice area (EOA) were evaluated. For histological analysis, AV leaflets sections were stained with Haematoxylin/Eosin and AlizarineRedS to highlight calcifications.

**Results:**

The treatment induced a reduction in ΔPsys in all tested samples, improving EOA, but caused an increase in backflow volume. Moreover, histology suggested AV leaflets integrity.

**Conclusions:**

The TDD procedure improved AV fluid-dynamics during systole in all tested samples, without evidence of damage to tissues. This suggests TDD could be a promising option to postpone AV replacement for patients with AVS.

## Introduction

Calcific aortic valve stenosis (AVS) is the most common valve disease in developed countries, affecting 12.4% of adults over the age of 75, with 3.4% of those presenting severe AVS [1]. Fibrosis and calcifications have a key role in the generation and progression of the disease. Fibrous tissue formation and calcium minerals accumulation within the valve leaflets and annulus can lead to thickening and stiffening of the aortic valve (AV) leaflets, causing severe obstruction of cardiac outflow. AVS progression is subtle, being frequently asymptomatic. Unfortunately, when symptoms appear, the risk of death increases dramatically.

Current guidelines recommend AV replacement in patients with severe AVS in the presence of symptoms or a left ventricular ejection fraction < 50% [2].

Open-heart Surgical Aortic Valve Replacement (SAVR) has been the first-line therapy for patients with severe symptomatic AVS. In such treatment, the stenotic native valve is replaced by an artificial one, which can be a mechanical or biological AV prosthesis. Over the last decades, the use of biological prostheses has significantly increased compared to mechanical ones, in all age groups [3]. Additionally, in recent years, the implantation of stent-mounted biological prostheses through a transcatheter approach (Transcatheter Aortic Valve Replacement - TAVR) has significantly grown, with a growing indication also in lower-risk patients [4], ensuring reduced invasiveness and shorter hospitalization periods.

However, both surgical and transcatheter bioprosthetic valves are prone to structural valve degeneration (SVD), an unavoidable process associated with calcification formation, which limits their durability [5, 6]. This increases the likelihood of a challenging re-intervention, especially for younger patients in whom SVD is accelerated due to a more pronounced immunologic response [7]. A recent systematic review has defined aortic bioprosthesis durability as the main concern in non-elderly patients [8].

Therefore, there is a need to explore alternative therapeutic solutions for all patients with severe calcific AVS with AV replacement indication. These options should aim to improve the prognosis and quality of life of the patient, as well as postpone prosthetic valve implantation or a second reoperation.

In this regard, transcatheter ultrasound-based decalcification procedures can represent an innovative solution in cardiovascular therapy. If properly modulated in intensity, frequency, and waveform, ultrasounds can be used to generate microbubbles inside the spongiosa, which, expanding and imploding cyclically, produce fractures and structural changes in calcific deposits [9].

With the aim of fragmenting calcium deposits and restoring leaflets flexibility and mobility, AorticLab S.r.l. (Italy) is developing the Transcatheter Debridement Device (TDD), a novel percutaneous device that generates ultrasonic pulse waves on calcific AV leaflets.

TDD has already been demonstrated to be effective in calcium deposits rupture in explanted human calcified leaflets [9, 10]. However, there are no studies that report on the effectiveness of this device in restoring AV hemodynamic performance.

In this work, we performed a preliminary ex vivo study on human hearts to quantify how TDD treatment affects stenotic AVs hemodynamic. Furthermore, qualitative histological analysis was carried out to assess if TDD treatment could alter or affect the structure of AV leaflets.

## Methods

### Transcatheter Debridement Device

The TDD device used for this test session was a scaled-down prototypal version of the transfemoral ones. However, it retains all the functional elements and movement controls of the full-sized device.

During the testing phase, commercial guidewires and catheters were not employed; only the prototypal TDD system was used. To perform the decalcification treatment, the device was deployed directly into the aortic root without navigating through the femoral route.

The TDD includes four functional modules:

1. an ablation unit, based on two piezoelectric transducers able to produce local low-intensity ultrasound shockwaves;
2. a silicone-stented artificial valve, designed to temporarily replace the AV function during the ablation procedure;
3. a delivery system that comprises a multi lumen transfemoral catheter, a handle, and the electrical connection wires;
4. a pulse generator to provide two impulsive electrical signals to the ablation unit.

**Fig. 1:**
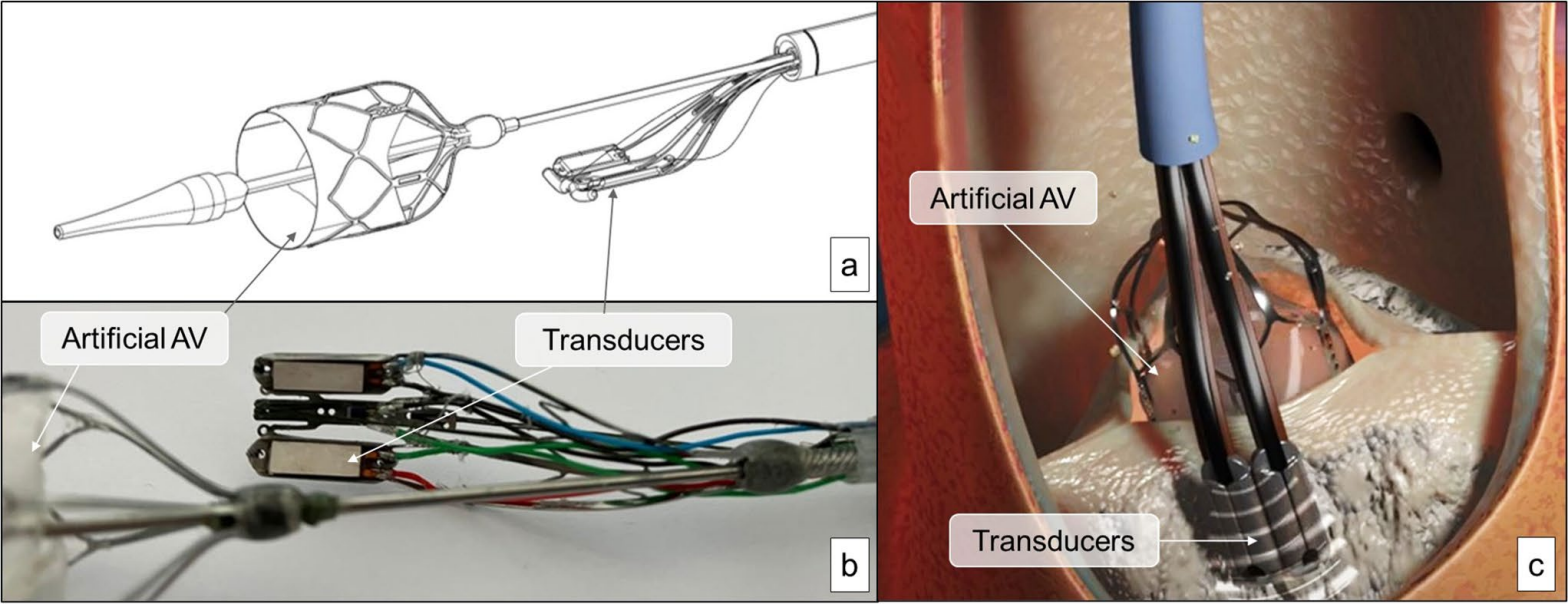
Transcatheter debridement device illustration. (**a**) Drawing of piezoceramic transducers and the artificial AV; (**b**) Photo of piezoceramic transducers; (**c**) Representation of TDD positioning.

The ablation unit is based on two piezoelectric transducers (PZTs) able to generate two localized low-intensity ultrasound shockwaves at frequencies of 150 kHz and 3125 kHz. These transducers are mounted on a nitinol structure that, being able to rotate 360 degrees, allows to focus the ablation on the chosen calcific leaflet to be treated (a, b, Fig. 1).

The TDD also includes a polymeric artificial valve that is positioned at the level of the native AV annulus during the debridement procedure. This valve is used to temporarily replace the AV function during the ablation (c, Fig. 1).

The device is delivered to the AV through the femoral artery using a delivery system. This system includes two multi-lumen catheters designed to carry electrical connections, as well as PZTs and the polymeric valve.

In the final section of the delivery system, a customized pulse generator is connected, which simultaneously outputs two pulse waves to the PZTs.

Ultrasonic fields induce the formation of microbubbles within leaflets calcifications. The sequential application of two shock waves at different frequencies triggers the cavitation phenomenon. During cavitation, the bubbles expand and contract, fragmenting the calcific deposits and modifying the fibrotic structure of the valve leaflets, thus restoring tissue pliability.

To mitigate the risk of embolization from calcific debris and reduce procedure-related strokes, TDD is designed to be used in combination with an embolic protection device.

Since the purpose of this study is to evaluate decalcification treatment’s effectiveness on AV hemodynamics, no tests were made on the delivery system or its usability.

### Heart Sample Preparation

Three human cadaveric hearts affected by AVS were tested at the “École de Chirurgie” in Lyon.

After pre-selection based on available clinical information, the heart samples were selected following an inspection by an experienced surgeon.

Within 48 h of death, corpses were collected at the anatomy laboratory. To preserve the bodies, a safe balm solution (400 mL per 3 L of solution) was injected into the carotid, subclavian, or femoral artery. Following the injection, the bodies were stored at a temperature of 2 °C.

Hearts were taken by thoracotomy from cadavers within two weeks after death, taking care to preserve the heart chambers and at least 3 cm of the annexed blood vessels. Samples, immersed in saline solution, were stored at -16.5 °C, and placed at 6 °C for 48 h before the day of testing.

Before tests, each heart sample was prepared as follows. The pulmonary veins and coronary arteries were closed with sutures. 3D printed custom-made hydraulic connectors were secured through 1” tubes to the left heart at the level of the left atrium (atrial connector), aorta (aorta connector), and through the heart apex (apical connector). The connectors were used to integrate the heart samples within an ex-vivo beating heart simulator.

### Ex-Vivo Beating Heart Simulator

Treatment of AVS using TDD was assessed in an ex-vivo beating heart simulator that was described in detail in a previous study [11].

**Fig. 2:**
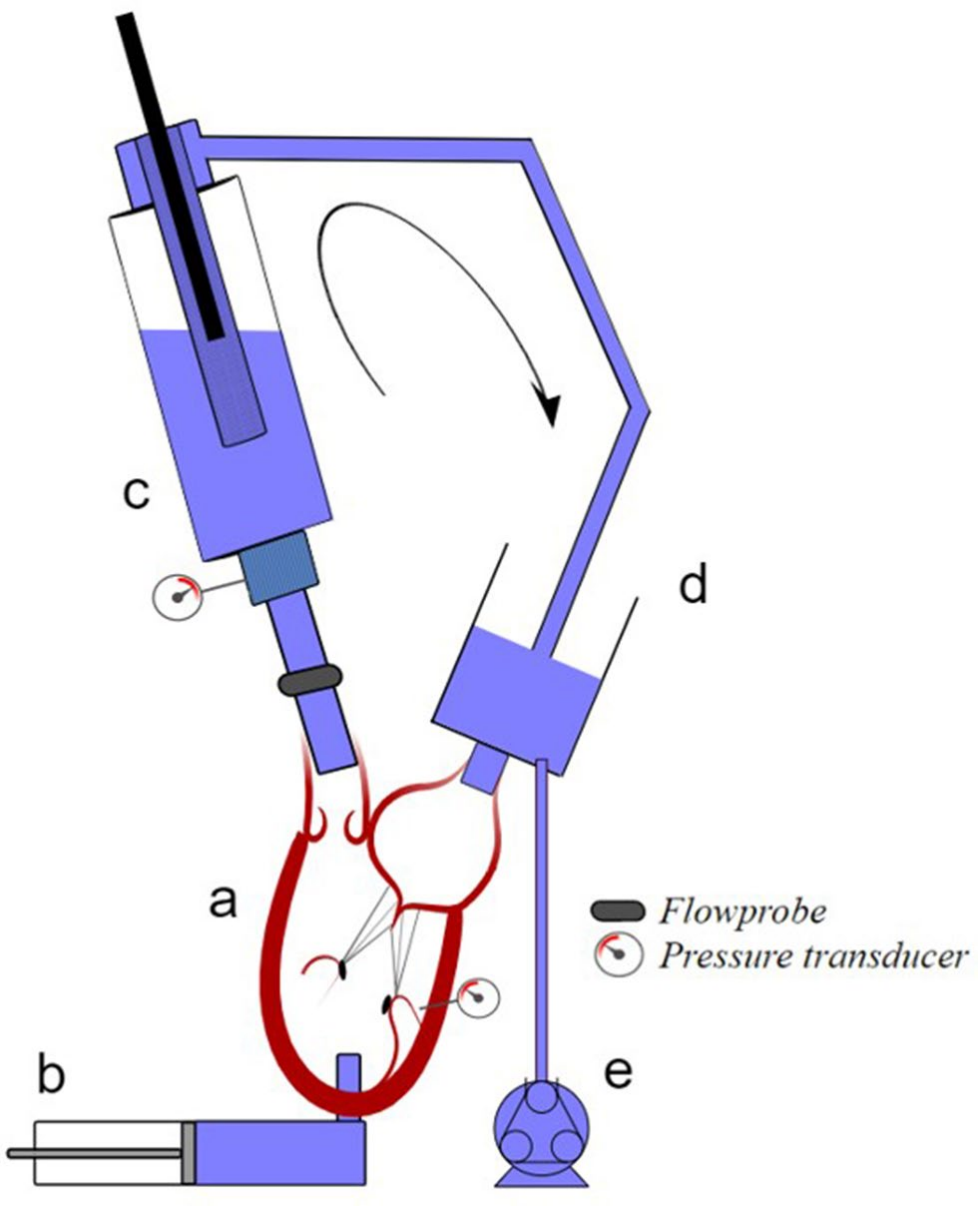
Scheme of the ex-vivo beating heart simulator. (**a**) left heart, (**b**) volumetric pump, (**c**) systemic impedance simulator, (**d**) preload reservoir; (**e**) roller pump. The arrow indicates the direction of the average flow rate.

A scheme of the setup is reported in Fig. 2. In brief, the ex-vivo beating heart simulator was actuated by a custom-made pulsatile pump (motor: MCS06C41, Lenze, Hameln, Germany; controller: Servo9322EK, Lenze; software: Global Drive Control 4.14, Lenze), (b, Fig. 2), which allowed to replicate the physiologic flow rates through the mitral and aortic valves [12]. The pulsatile pump was connected to the left ventricle of the heart (a, Fig. 2) through the apical connector. The aortic root was connected through the aortic connector to a systemic impedance simulator (c, Fig. 2), that simulated the hydraulic systemic input impedance. The outflow of the impedance simulator closed the hydraulic circuit through 1/2” tube in a preload reservoir (d, Fig. 2), connected to the left atrium through the atrial connector. A roller pump (Sorin Stockert Shiley 10-10-00, Stockert Instrumente, Munchen, Germany) (e, Fig. 2), connected to the reservoir with 1/4” tube, was used to fill the circuit with water at room temperature.

Pulsatile flow working conditions were set as follows. Heart rate (HR) of 60 bpm was set to reproduce typical rest conditions; mean aortic pressure (AoP) was limited to 50 mmHg, to preserve the heart tissues; and pump stroke volume was adjusted for each sample to obtain a comparable effective stroke volume between the pre- and post-treatment conditions.

Aortic flow rate was measured with a transit-time flowmeter (HT110R, Transonic System, Inc., Ithaca, NY, USA), equipped with a 1″ probe placed downstream the AV (Fig. 2). Systemic and ventricular pressures were measured by piezoresistive transducers (143PC05D model, 140PC series, Honeywell, Inc., Morristown, NJ, USA) placed downstream the AV and on the left ventricular wall, respectively (Fig. 2). All signals were acquired with an A/D converter (DAQ USB 6210, National Instruments, Austin, TX, USA) at a sampling frequency of 200 Hz. An endoscope (Karl Storz Nova 201315 20, 300Watts Xenon Light), equipped with a 30° lens (HOPKINS), was used to directly inspect the kinematics of the AV and the entire ablation procedure.

### Test Protocol

Each heart sample underwent hemodynamic characterization under pulsatile flow condition before treatment (baseline).

Treatment with TDD was then performed as follows. The pulsatile pump was turned off, and the aortic connector was detached from the systemic impedance simulator.

The leaflets to be treated were selected by an experienced surgeon through a visual inspection and an assessment of the leaflet’s residual mobility. Except for heart Sample 2, where two leaflets were treated, one leaflet per heart was chosen for the TDD treatment to minimize procedure times.

**Fig. 3:**
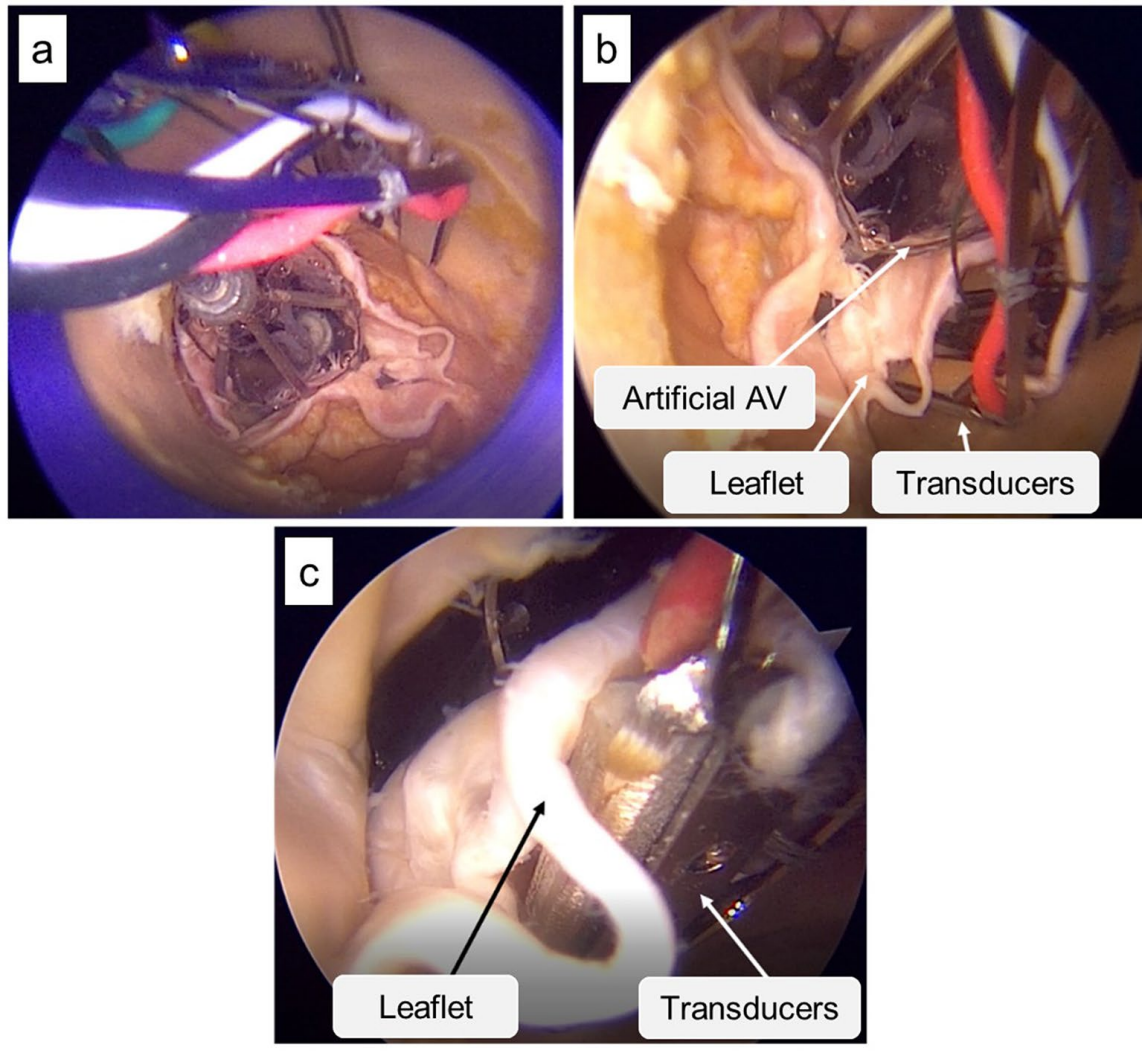
Endoscopic images of TDD positioning. (**a**) Deployment of valve and transducers, (**b**) Positioning of the transducers near the chosen leaflet, (**c**) Coupling of the transducers with the leaflet.

The TDD delivery system was passed through the AV. Once the catheter tip was approximately 3 cm beyond the AV annulus, the external shell of the system was retracted, allowing the polymeric valve to be deployed within the native AV leaflets. The piezoceramic transducers were then positioned in contact with the calcium deposits of the chosen AV leaflet. Using the handle of the delivery system, the transducers were adjusted to clamp the tissue to be treated between the artificial valve and themselves. Finally, TDD was activated (Fig. 3).

To ensure ultrasound transmission, the AV was kept constantly immersed in water during the ablation procedure.

The ablation treatment was performed continuously for 30 min. Impulsive low-intensity ultrasound shock waves were emitted at two different frequencies (3125 kHz and 150 kHz) in an alternating pattern with a time interval of 6 s.

During the entire procedure, the water temperature in the ablation site was monitored every 5 min by a thermistor placed on TDD system (RTD-Pt100 thermistor, compliant with IEC 60751) to ensure the device was not generating heat that might damage tissues; moreover, the TDD device positioning was inspected with the endoscope.

After treatment, the heart was reconnected to the pulsatile system, and post-treatment hemodynamic assessment was performed.

### Data Processing

The raw hemodynamic data was averaged over 10 cardiac cycles and the following indexes were derived:

- Mean aortic pressure (AoP, in mmHg);
- Stroke Volume (SV, in mL);
- Mean systolic pressure drop across AV (ΔPsys, in mmHg);
- Effective Orefice Area (EOA, in cm^2^)– calculated as follows [13]:

$$EOA=\frac{{{q_{vRMS}}}}{{51.6*\sqrt {\frac{{\Delta {P_{sys}}}}{\rho }} }}$$

where q_vRMS_ is the root mean square forward flow (ml/s) during the positive transvalvular pressure period, ΔP_sys_ is the mean pressure difference measured during the positive differential pressure period (mmHg), ρ is the density of the test fluid (g/cm^3^).

- Backflow volume (BV, in mL)– integral of the negative portion of aortic flow curve.

### Preliminary Histological Analysis

Qualitative histological analysis was performed to examine AV leaflets after TDD treatment.

Following the hemodynamic tests, the aortic root of each heart sample was removed and preserved in formalin 4%.

Both the inflow and outflow surfaces of all AV leaflets (treated and untreated) were observed and photographed using the stereoscopic microscope Leica Z6 APO.

After the macroscopic examination, all leaflets of analysed valves were totally resin embedded and the face of inclusion coincides with the free edge of the leaflet.

For each leaflet, five histological Sect. (4–5µ in thickness) were performed to analyse the entirety of the leaflet.

All sections were stained with Haematoxylin/Eosin, to evidence presence of potential structural alterations and inflammatory cells, and Alizarine Red S, to highlight the calcium deposits.

Each slide was observed with a biological microscope (Nikon Eclipse E600, Nikon Instruments Inc, Melville, N.Y., U.S.A.) and photographed (Leica DFC450), adding a dimensional marker. The photographic documentation was performed at low and high magnification on significant slides.

## Results

AV morphological and hemodynamic alterations were observed after the TDD treatment.

**Fig. 4:**
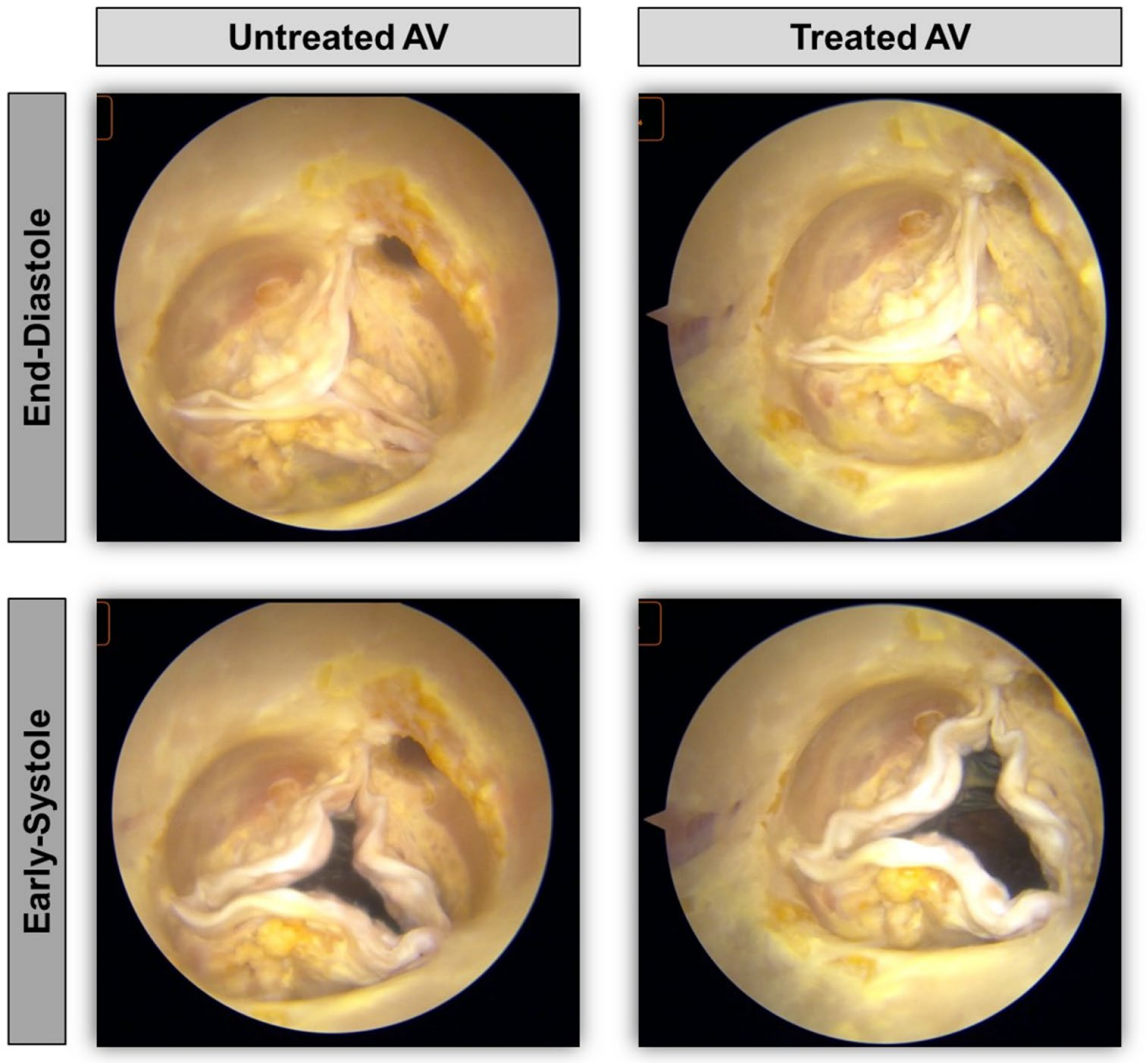
Endoscopic images of Sample 3 aortic valve (AV). Untreated (left) and treated (right) AV at end-diastole (top) and early-systole (bottom) phases.

Fig. 4 shows an example of AV endoscopic images recorded for a sample before and after treatment.

In all tested samples, at the beginning of TDD procedure, the water temperature at the ablation site was around 25.95 °C. A maximum temperature of 26.4 °C was reached 30 min after treatment started in Sample 2.

### Hemodynamic Assessment

**Fig. 5:**
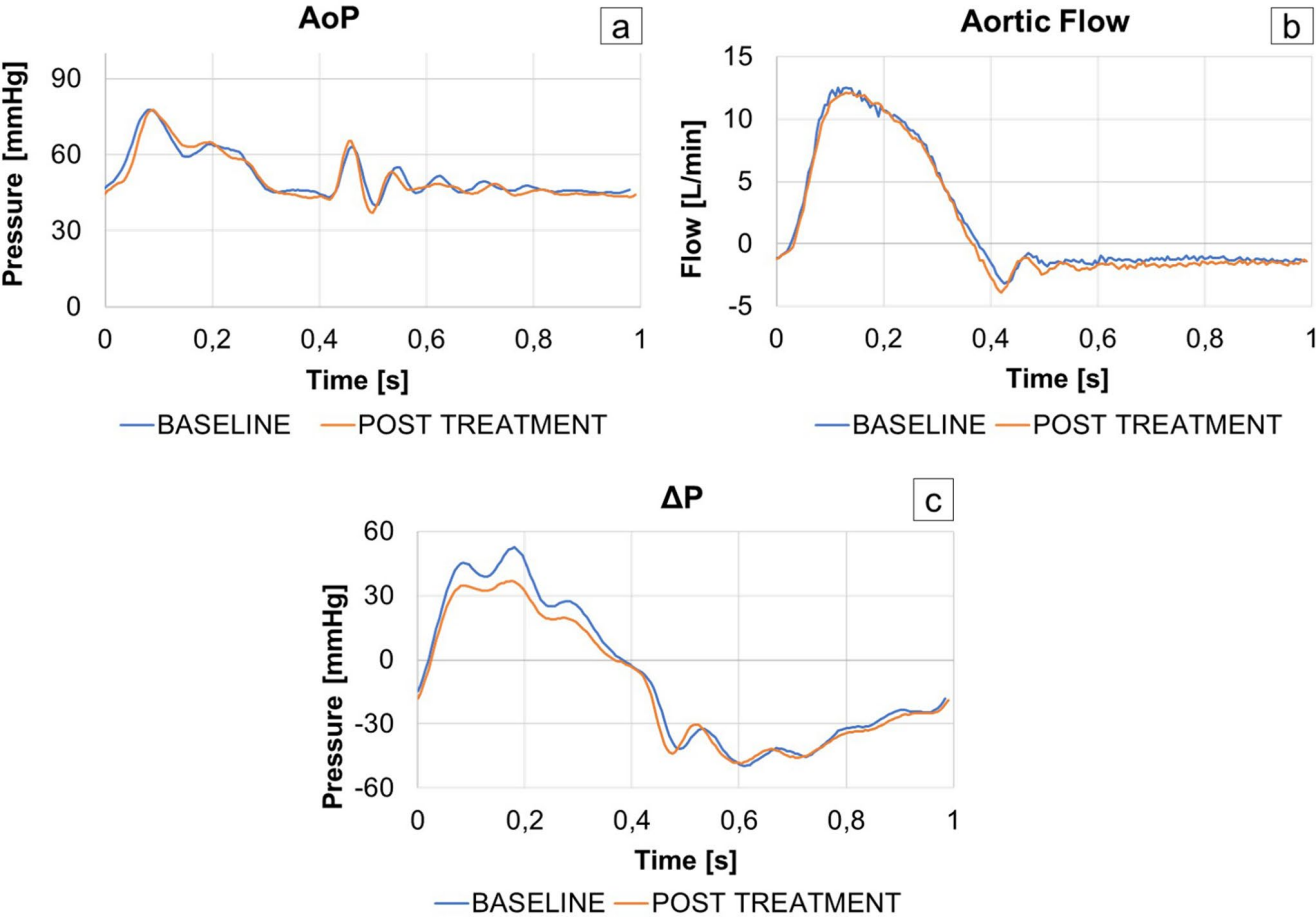
Experimentally obtained aortic pressure (**a**), aortic flow (**b**) and transvalvular pressure (**c**) curves in baseline (blue) and post-treatment (orange) condition for the same heart sample (Sample 3).

Figure 5 shows an example of aortic pressure, transvalvular pressure and aortic flow signals recorded for a sample in baseline and post-treatment conditions. Table 1 shows the treated leaflets and the analyzed hemodynamic parameters for each sample. Data were presented as mean ± standard deviation, where applicable.

**Table 1:** – Treated leaflets for each heart sample and fluid dynamic indexes calculated.

	Sample1		Sample2		Sample3	
	Baseline	Post treatment	Baseline	Post treatment	Baseline	Post treatment
***Treated leaflets***	RCL		NCL and RCL		RCL	
***Conditions***						
AoP [mmHg]	50,15 ± 0,05	50,08 ± 0,03	51,83 ± 0,33	50,90 ± 0,22	51,77 ± 0,41	50,82 ± 0,07
SV [ml]	40,26 ± 0,29	40,20 ± 0,17	31,34 ± 1,09	32,66 ± 0,69	45,57 ± 0,57	43,67 ± 0,44
***Systole***						
ΔPsys [mmHg]	6,03 ± 0,06	4,44 ± 0,12	13,35 ± 0,50	12,46 ± 0,10	29,72 ± 0,10	23,35 ± 0,19
EOA [cm^2^]	1,17 ± 0,01	1,39 ± 0,02	0,58 ± 0,01	0,63 ± 0,01	0,51 ± 0,01	0,58 ± 0,01
***Diastole***						
BV [ml]	9,23 ± 1,06	18,25 ± 1,28	15,75 ± 0,90	19,37 ± 1,29	14,31 ± 1,81	18,84 ± 1,33
ΔPsys variation [%]	-26%		-7%		-21%	
EOA variation [%]	18%		9%		14%	
BV variation [%]	98%		23%		32%	

RCL, right coronary leaflet; NCL, not coronary leaflet; AoP [mmHg], mean aortic pressure; SV [ml], stroke volume calculates as integral of the positive aortic flow curve; CO [l/min], cardiac output calculates as average of the aortic flow curve; ΔPsys [mmHg], mean systolic pressure drop across AV; EOA [cm^2^], effective Orefice Area; BV [ml], backflow volume calculates as integral of the negative aortic flow curve; ΔPsys variation [%]; EOA variation [%]; BV variation [%]. Data are presented as mean ± standard deviation

### Working Conditions Assessment

The tests were performed under controlled pressure and flow conditions, as shown in Fig. 5, a-b. The AoP was set in a reproducible manner to a mean value of 50.92 ± 0.75 mmHg. Similarly, the SV of each sample was highly comparable between baseline and post-treatment, with maximum difference of 1.9 mL/cycle.

### Performance Indexes Assessment

The TDD treatment induced improvement in ΔPsys and EOA in all treated samples. A reduction in ΔPsys was observed between baseline and post-treatment condition in each sample, with an associated increase in EOA. Additionally, an increase in BV values was noted following the treatment.

### Histological Assessment

After the fluid dynamics tests, a qualitative histological analysis of treated and untreated leaflets was carried out.

**Fig. 6:**
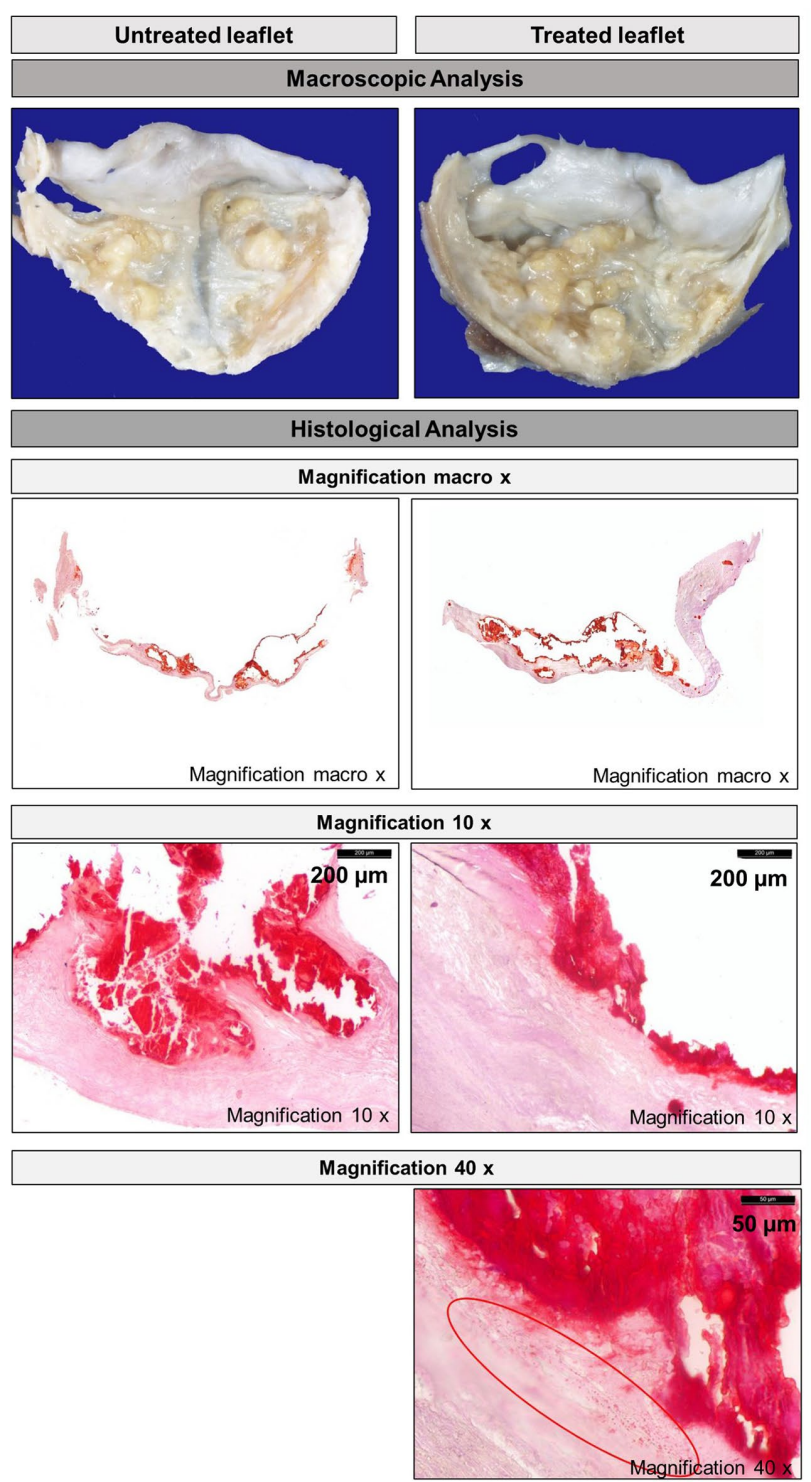
Macroscopical analysis and histological analysis (stain Alyzarine Red S) of heart Sample 2. Increasing magnifications of the same section of untreated (left) and treated (right) leaflet. Calcium debris, probably due to decalcification treatment, are circled in red.

The macroscopic examinations highlighted the presence of large intrinsic and vegetating calcifications in all leaflets (treated and untreated). Both the ventricular and aortic sides of all leaflets were locally wrinkled and showed yellowish areas due to calcifications. Deep folds were present in all leaflets due to their altered movement. Only in some treated leaflets, there were scars probably due to the positioning procedure (Sample 2 leaflets). Figure 6 shows an example of macroscopical images of untreated and treated leaflets.

From the histological analysis, all leaflets (untreated and treated) showed structural alterations, inflammatory cells, and large intrinsic calcifications due to the degeneration of the AV during the patient’s life. Furthermore, in all treated leaflets fragmentation of the calcium deposits was visible, possibly caused by the ablation process.

Fig. 6 shows an example of histological images of untreated and treated leaflets at increasing magnifications. Calcium debris, probably due to decalcification treatment, are circled in red at magnification 40x.

## Discussion

In an aging world, there is an increasing need to delay valve replacement in young patients and to avoid reoperation in high-risk patients. TDD aims to fragment calcium deposits and restore leaflets’ mobility of degenerated valves by exploiting ultrasonic pulse waves, postponing prosthesis implantation or reimplantation, and improving patients’ prognosis.

Bioprosthetic surgical and transcatheter AV replacements represent the current standard options for the treatment of calcific AVS. The non-requirement of a life-threatening anticoagulation therapy makes them preferable to the mechanical prostheses. Despite biological valves demonstrate excellent hemodynamic properties similar to those of native valves, they are subjected to structural degeneration, an irreversible prosses associated with calcification formation, which limits their durability [14]. This increases the risk of a re-intervention, especially for younger patients in whom SVD is accelerated due to a more pronounced immunologic response [15].

Given that both surgical and transcatheter bioprosthetic valves have a limited lifespan, it is crucial to delay prosthesis implantation to minimize the number of valve replacement a patient may need over their lifetime, thereby improving their prognosis.

TDD represents a new strategy that could bring potential advantages in the current treatment process of AVS. The system fragments and removes calcifications from the AV leaflets, improving the functionality of the degenerated native or prosthetic valve without requiring the implant of any permanent artificial device. This postpones the implantation of a prosthesis and possible related complications. Furthermore, due to its minimally invasive nature, the TDD procedure could reduce the length of hospital stay, accelerate recovery times, and reduce overall health costs compared to traditional surgical methods, and ideally also compared to TAVR. The idea of performing n-number of TDD treatments on the same degenerated valve, increasing its longevity, makes TDD a promising strategy to delay AV replacement for patients with calcific AVS.

In this study, we performed a preliminary evaluation of this new debridement device for the treatment of calcific and fibrotic valves. To evaluate the impact of the TDD on AV hemodynamics, we performed an ex vivo investigation on human hearts with AVS. Moreover, qualitative histological analyses on a macro- and micro-scale were conducted to examine the structural integrity of the AV after TDD treatment.

We evaluated the TDD procedure by characterizing each heart sample under pulsatile flow conditions before and after treatment, investigating AV flowrate and transvalvular pressure alterations. Our investigation showed that TDD treatment improves AV fluid dynamics during systole in all tested samples, without evidence of damage to AV tissues.

Our ex-vivo beating heart simulator proved to be suitable for the intended purpose of our research. The use of human cadaveric samples affected by AVS provided a pathological model ready to use and realistic in terms of calcifications morphology and composition. This approach eliminated the need for lengthy preparation times required by other strategies, that exploit chemical accelerators [16] or human calcific leaflets [17] sutured on porcine heart models. Moreover, the beating simulator allowed us to conduct the experiments under highly controlled and repeatable working conditions, enabling us to make a fair comparison between pre- and post-treatment conditions and draw inferences from the few tested samples.

The results pointed out that TDD treatment had a noticeable effect on AV hemodynamics. In all treated samples, we observed a reduction in systolic transvalvular pressure drop with a consequent increase in EOA. This result indicates that the TDD treatment effectively improved the mobility of the leaflets. However, it is important to note that Sample 2 showed a poor improvement in systolic transvalvular pressure, despite two leaflets being treated with TDD. This could probably be due to the bulky calcium deposits present on the AV leaflets. TDD has been shown to have limitations in ablating calcifications above a certain volume [18]. Therefore, further studies are needed to assess how different morphologies, volumes, and locations of calcifications may affect treatment efficacy.

The outcomes indicate that the TDD had an influence on valve closure as well, with an increase in backflow volume in all tested samples. This result could be attributed to the ablation procedure, which may have changed the reciprocal arrangement of the AV leaflets, altering their coaptation and resulting in suboptimal valve closure. This indicates that the choice of the site for treatment, affecting leaflet mobility, could be crucial to achieve improvement in hemodynamic performance of the valve, both in systole and diastole. In this perspective, specific studies are needed.

Endoscope videos recorded following the TDD procedure showed no major tissue damage. These findings were supported by qualitative histological analyses, which confirmed the integrity and structural coherence of the AV tissue post-TDD treatment. Furthermore, fragmentation of the calcium deposits observed in all treated leaflets confirmed the effectiveness of the ablation process.

In contrast with the design approach of delivering the device through the femoral artery to the landing zone, in this study the TDD transducers were directly placed on the calcific leaflet, this allowing for a precise and repeatable positioning. This approach simplified the procedure and minimized possible confounding factors, enabling a focused analysis on the device’s physical principle rather than the procedural aspects. This strategy was in line with the study’s rationale, which aimed only to assess the effectiveness of the decalcification treatment on the AV hemodynamics without considering the device’s delivery system or its usability. Preliminary usability and navigability in vivo animal tests have already been performed, using a porcine model [9]. However, delivery approach and technology, as well as the repeatability of the device deployment in the appropriate anatomical district, must be accurately assessed in ad hoc-designed studies.

The study has several limits. One major limitation was the small number of hearts, due to the poor availability of pathologic cadaveric hearts. The number of tested samples prevented from performing a robust statistical analysis. Moreover, the advanced age of the donors made the tissues of the tested samples very fragile, preventing from setting physiological working conditions during the tests. It is also of note that the TDD procedure was performed under static conditions with the pulsatile pump turned off during treatment. A first preliminary test in beating-heart condition was described in detail in a previous study [9], but further tests using calcific models are necessary to assess the stability of the positioning during TDD treatment.

These limitations highlight the need for further research with a larger number of samples and an evaluation under fluid dynamic physiological conditions. Moreover, the dependence of the effectiveness of the treatment on calcium deposits shapes must be accurately evaluated.

However, this study could be considered a preliminary demonstration of the Transcatheter Debridement Device’ capability to potentially improve calcified AV hemodynamic without compromising the AV structure. TDD is a promising option to delay AV replacement for patients with AVS.
